# Peptide Vaccine: Progress and Challenges

**DOI:** 10.3390/vaccines2030515

**Published:** 2014-07-02

**Authors:** Weidang Li, Medha D. Joshi, Smita Singhania, Kyle H. Ramsey, Ashlesh K. Murthy

**Affiliations:** 1 Department of Pathology, Midwestern University, Downers Grove, IL 60515, USA; E-Mail: wli@midwestern.edu; 2 Department of Pharmaceutical Sciences, Midwestern University, Downers Grove, IL 60515, USA; E-Mail: mjoshi@midwestern.edu; 3 Symbiosis School of Biomedical Sciences, Symbiosis International University, Pune, MH 412115, India; E-Mail: smita.singhania@gmail.com; 4 Department of Microbiology and Immunology, Midwestern University, Downers Grove, IL 60515, USA; E-Mail: kramse@midwestern.edu

**Keywords:** peptide vaccine, epitope, adjuvants

## Abstract

Conventional vaccine strategies have been highly efficacious for several decades in reducing mortality and morbidity due to infectious diseases. The bane of conventional vaccines, such as those that include whole organisms or large proteins, appear to be the inclusion of unnecessary antigenic load that, not only contributes little to the protective immune response, but complicates the situation by inducing allergenic and/or reactogenic responses. Peptide vaccines are an attractive alternative strategy that relies on usage of short peptide fragments to engineer the induction of highly targeted immune responses, consequently avoiding allergenic and/or reactogenic sequences. Conversely, peptide vaccines used in isolation are often weakly immunogenic and require particulate carriers for delivery and adjuvanting. In this article, we discuss the specific advantages and considerations in targeted induction of immune responses by peptide vaccines and progresses in the development of such vaccines against various diseases. Additionally, we also discuss the development of particulate carrier strategies and the inherent challenges with regard to safety when combining such technologies with peptide vaccines.

## 1. Introduction

Vaccination has proven to be the mainstay in prevention of various deadly infectious diseases [1,2,3]. Historically, live-attenuated or inactivated forms of microbial pathogens (viruses, bacteriae, *etc.*) have been used for induction of antigen-specific responses that protect the host against subsequent infections. Based on the pathogen being used, such vaccine formulations can contain anywhere between tens of to a few hundred proteins. However, protective immunity is usually dependent upon a few select proteins within such formulations, whereas the majority of proteins are unnecessary for the induction of protective immunity. Furthermore, these additional proteins may induce allergenic and/or reactogenic responses, thus emphasizing the need to eliminate them from vaccine formulations. This rationale led to an interest in subunit vaccines using single, or a select few, proteins of the microbes in vaccine formulations for induction of protective immunity [4,5]. An extension of this logic would be that even single proteins contain many hundreds of antigenic epitopes, all of which are not necessary; whereas some may even be detrimental to the induction of protective immunity. This has created an interest in “peptide vaccines” containing only epitopes capable of inducing positive, desirable T cell and B cell mediated immune response [6]. “Peptides” used in these vaccines are 20–30 amino acid sequences that are synthesized to form an immunogenic peptide molecule representing the specific epitope of an antigen. On the one hand, since epitopes are the antigenic determinants within larger proteins, these peptides are considered sufficient for activation of the appropriate cellular and humoral responses [7,8], while eliminating allergenic and/or reactogenic responses. Additionally, peptide vaccines can be used for induction of broad-spectrum immunity against multiple serological variants (serovars) or strains of a given pathogen by formulating multiple non-contiguous immunodominant epitopes and/or epitopes conserved between different serovars/strains of a pathogen. On the other hand, owing to the relatively small size of peptides, they are often weakly immunogenic by themselves and therefore require carrier molecules, to add chemical stability and adjuvanting, for the induction of a robust immune response [9,10]. Allergenicity and/or reactogenicity of carrier molecules themselves increase the complexity of peptide vaccine design. Manufacturing of peptide vaccines is generally considered as safe and cost effective when compared to conventional vaccines. There are many peptide vaccines under development, such as vaccine for human immunodeficiency virus (HIV) [11], hepatitis C virus (HCV) [12], malaria [13], foot and mouth disease [14], swine fever [15], influenza [16], anthrax [17], human papilloma virus (HPV) [18], therapeutic anti-cancer vaccines [19,20,21,22,23,24] for pancreatic cancer, melanoma, non-small cell lung cancer, advanced hepatocellular carcinoma cutaneous T-cell lymphoma and B-Cell chronic lymphocytic leukemia. 

A database of publicly and privately conducted clinical studies is maintained on ClinicalTrials.gov, which is a service of the U.S. National Institute of Health. Around 452 clinical studies of peptide vaccines for preventive or therapeutic purpose on multiple disease conditions are registered with this database until mid-March 2014 (Table 1). The vast majority of candidate peptide vaccines are under Phase I (270 studies) and Phase II (224 studies) stage of development. In a total of 452 studies, only 12 studies have progressed to Phase III level of development. Interestingly, all these 12 studies are on therapeutic candidate peptide vaccines indicated for treatment of multiple types of cancers. Preventive or therapeutic candidate peptide vaccines for infectious diseases such as HIV, HCV, Hepatitis B virus (HBV), Cytomegalovirus (CMV), Influenza, Tuberculosis, Malaria, Pneumonia, Genital Herpes, Hand Foot and Mouth disease, and also for Allergy (Cat allergy, Ragweed allergy, Grass allergy) House dust mites—Rhinoconjuctivitis, Asthma, Diabetes, Alzheimer’s disease, are either under Phase II or Phase I stage of development. However, the commercialization of a licensed peptide vaccine has yet to be realized. 

**Table 1:** Worldwide clinical trials of peptide vaccines.

S.N.	Peptide Vaccines Under Development		Clinical Indications for Candidate Peptide Vaccines Under Development
1	No. of Phase I Studies	270	Anticancer studies, Malaria, Falciparum Malaria, Anti-Plasmodium vivax, Influenza, Alzheimer’s disease, Insulin dependent diabetes mellitus, Hand foot and mouth disease, anti HIV, HCV, HBV, CMV, Diabetes Mellitus, Type One, Cat allergy, Allergy
2	No. of Phase II Studies	224	Anticancer studies, anti HIV, HCV, HBV, CMV, Pneumococcal, genital Herpes—Herpes Simplex Type II, Tuberculosis, Diabetes, Diabetes Mellitus, Type One, Cat allergy, Ragweed allergy, Grass allergy, Ashtma, House dust mites - Rhinoconjuctivitis, maximum studies–anticancer
3	No. of Phase III Studies	12	Anti-cancer studies
4	No. of Phase IV Studies	NIL	No peptide vaccine reached market yet

## 2. Considerations and Methods for the Design of Peptide Vaccines

A variety of considerations need to be made during the design of a peptide vaccine, in context of the particular vaccine under development. First and foremost among them is the identification of immuno-dominant domains of epitopes that are capable of inducing protective immune response in terms of humoral immunity and/or cell mediated immunity against desired antigen [25]. Immunodominant epitopes can be chosen in context of B cells, cytotoxic or helper T cells. For example, one of the most suited approaches is preparation of protective monoclonal antibody against the conserved regions, which may be helpful in designing protective as well as therapeutic vaccine against cancers [26]. For such a vaccine, the selection of immunodominant B cell epitopes is important. On the other hand, vaccines against intracellular pathogens such as viruses or against cancers may focus on the identification of epitopes that induce cytotoxic T cell responses [20,27]. It is to be noted that for efficient induction of either B-cell or cytotoxic T cell responses, the induction of a robust helper T cell responses is crucial [27,28]. Thus, epitope selection will have to be directed towards not only inducing the required effector response (B cells or cytotoxic T cells), but also for induction of helper T cell responses. Among the epitopes that induce specific subsets of immune responses, the next challenge would be to identify the right epitope(s) or peptide(s) that can activate T cells to the magnitude that can confer protective immunity. Additionally, for development of a broad-spectrum vaccine against multiple serovars of a pathogen, it may be necessary to identify highly conserved immunodominant epitopes. Another issue to be considered is the processing, presentation and association of the candidate peptide vaccine by antigen presenting cells T cells in a highly MHC-heterogenous human population. 

Multiple biochemical and cellular immuno assays have been designed and utilized for selection of candidate peptides for vaccines. Various strategies employing either *in silico* approaches or experimental approaches, or combinations of both have been followed. The inherent complexity and cost of using experimental approaches at initial stages of screening has led researchers to seek the support of reliable and cost-effective bioinformatics *in silico* tools. A variety of bioinformatics tools are used for prediction including, but not limited to, the translocation of peptides into endoplasmic reticulum (MHC-I), cleavage in lysosomal compartments (MHC-II), binding of antigen to MHC I and MHC II, HLA haplotype specificity, and recognition by T cell receptors. Multiple epitope predictive algorithms have been developed as briefly described below:

(1) Structural resolution of desired antigen and its monoclonal antibody complex using nuclear magnetic resonance and X-ray crystallography to identify interactions at atomic level [29];
(2) Mass Spectrometry for the identification of monoclonal antibody binding antigenic epitope, and then using *in silico* techniques mapping them on the whole antigen to describe structure and sequence of the epitope [30]. Such computational analysis is usually done by first excluding antigen non-binding regions, and subsequently mapping the amino acid residues of the antigen identified by mass spectrophotometry analysis and the crystal structure;
(3) “Mimotopes” are peptides mimicking antigenic conformational structures that are recognized by paratope antibody. This is usually achieved by first generating a specific phage display library [31]. The identified peptides are then aligned to antigen sequence and subsequently superimposed to its 3D structure using *in silico* tools. An alternative approach is to express antigenic peptide from recombinant cDNA library and then screen for binding to specific monoclonal antibody. Using *in silico* tools, the selected peptide antigens can be further sequenced and aligned with antigen sequence, and if available, 3D structures can be superimposed. Some algorithms that can be of use are MimoPro, Mimox, Pepitope, MimoDB 2.0 [32];
(4) Prediction of linear B cell epitopes using computerized algorithms such as propensity scale, machine-learning algorithm or a combination of these two, hybrid algorithm, ABCpred, ANN-, BepriPred, HMM or more advanced algorithms are BEDDPRo, SVM, PSSM *etc.* [33,34];
(5) Usage of databases containing known T cell epitopes or peptides including information of their respective MHC binding and affinity of binding, the antigens involved in various clinical conditions, HLA restriction, host specificity, primary sequence of antigen *etc*. In case of development of peptide vaccines, selecting the correct target MHC or HLA is very critical, as the vaccine candidate should bind majority of HLAs in the population [35]. IMGT HLA database, which gives information of MHC alleles and its polymorphism and distribution in the community, is useful for this purpose [36]. A variety of structure-based algorithms also are available for *in silico* prediction of T cell epitopes. They are roughly classified as homology modeling, protein threading, and protein-protein docking. Prediction of conformational epitopes can be done using sequence, structure based, or binding matrices in silico algorithms such as DiscoTope, CEP, EPCES, PEPITO, SEPPA, EPSVR, ElliPro, BLAST-MODELLER, Epitpopia, CBTOPE, BEEPro, IEDB, SYFPEITHI, BIMAS, SMM, ANN, HMMs, SVMs, PROPRED, NetChop-3.0, *etc.* [36,37,38,39].

The identification, selection, and construction of candidate epitope(s) or peptide vaccine antigen(s) is followed by chemical synthesis of antigenic peptides. The synthesized peptides are subsequently conjugated to carrier molecules or adjuvants, as required. Immunoprofiling of resultant constructs is conducted *in vitro*, as well as in suitable animal models for determination of safety and efficacy, followed by progression to pre-clinical and clinical trials.

## 3. Induction of Protective T-Cell and B-Cell Mediated Immunity by Peptide Vaccines

A successful vaccine must induce a strong and long memory humoral and cellular immune response, but more importantly, protect against the disease being targeted. Therefore, it is important to evaluate whether the peptide immunogen can induce “protective” T-cell and B-cell immunity. The earliest peptide vaccination study came from virus-derived CD8 T-cell epitopes, which was reported by the late 1980s, and identified that mice vaccinated with small synthetically peptides can be recognized by CD8 cytotoxic T lymphocytes (CTL) [6,40,41,42,43,44]. The results also showed that those peptides were presented to MHC-I molecules *in vivo*, and consequently able to effectively induce protective T-cell responses that were able to resist a subsequent relevant virus challenge. In later studies, a number of synthetic peptides from influenza, LCMV, and Sendai virus were tested in different groups [45,46,47]; many of them have been successfully shown to induce specific CD8 cytotoxic T lymphocytes in immunized animals. Peptides recognized by CD8 T cells have been shown to be both selective and extremely sensitive; one amino acid change can alter the specific epitope into a non-immunogenic peptide [17,28,33]. The first synthetic peptide vaccine providing protection came from studies of canine parvovirus in dogs; protection was just as efficacious as the classical vaccine prepared from whole virus [34].Other studies have also shown that the protection mediated by some of these peptide vaccines occurs in a distinct T-cell mediated fashion, without the requirement for neutralizing antibody [1]. 

Since specific CD8 T cell-mediated immunity also plays a central role in controlling tumor growth, peptide-based vaccines also have been designed to use for tumor therapeutic applications. There have been several reports of peptides vaccination being successful in controlling tumor growth in mouse models [6,23,48,49]. In most of those studies, the synthetic peptides used were often longer than the 9–11 amino acids of the minimal peptide-sequence recognized by CD8 T cells. The longer peptides need to be trimmed to minimal MHC-I binding ligands by proteases and peptidases or by professional Antigen Presenting Cells (APC) process, followed by loading onto MHC-I groves. In fact, minimal peptides used in most of studies induce lower immune responses compared to longer peptides [50,51], since they only elicit CD8 T cell response without processing by APC. For example, Bijker *et al.*, demonstrated that long peptides need uptake and processing before the minimal epitope is able to bind to MHC-I molecule, and they can induce sustained CD8 CTL responses. Vaccination with long peptides resulted in somewhat delayed but sustained CTL response [7]. Kenter *et al.*, also proved long peptide vaccination against HPV 16 E6/E7 is superior over vaccination with short peptides, an aspect confirmed by later studies [52]. This is most likely the result of an insufficient effector CD8 T cell response due to the absence of stimulation of professional APC and memory T helper cell elicitation. 

**Figure 1:**
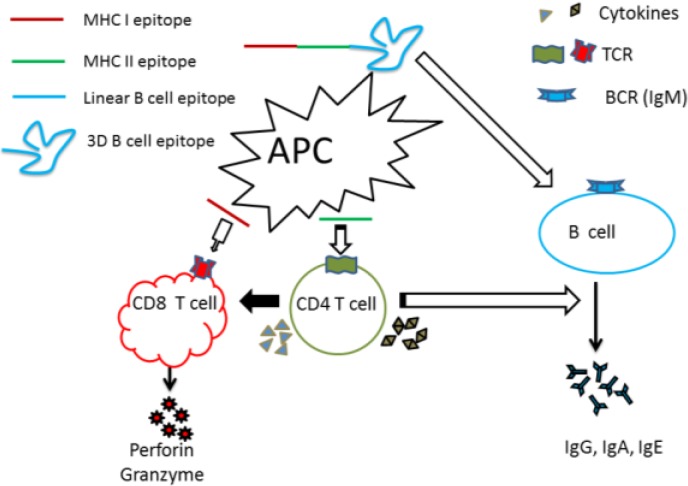
Central role of CD4^+^ T cells in peptide vaccines. Vaccine-induced immune response to control microbial pathogens may involve cytotoxic CD8^+^ T cell responses, helper CD4^+^ T cell responses, or antibody (B cell) responses. T cells recognize linear epitopes presented by antigen presenting cells, whereas B cells are capable of recognizing linear and conformational epitopes on soluble antigens. The induction of robust CD8^+^ T cell and/or antibody responses requires cytokine help from CD4^+^ T cells. Therefore, regardless of the nature of protective immune response required, the induction of CD4^+^ T cell responses is critical.

The impact of CD4 helper T cells in peptide vaccination was proven by subsequent studies in many model systems, and has been depicted in Figure 1. For example, the LCMV 15-mer synthetic long peptide vaccination study revealed that the effectiveness of LCMV 15-mer peptides to induce LCMV-specific CD8 T cell reactivity depended on CD4 T-cell help, suggesting that these 15-mer peptides comprised a helper epitope [53,54]. Similar results came from a HPV16 E7 peptide vaccination study in which the HPV16 E7 peptide vaccine in Freund’s incomplete adjuvant (FIA) failed to induce strong peptide-specific CD8 T-cell response in MHC-II knockout mice. This peptide comprised a T helper epitope that overlaps with the CTL epitope [55,56,57,58]. These data suggest that activation of antigen specific helper cells by peptide, comprising helper-epitopes, is very important for development of peptide vaccines, and the generation of protective CD8 T cell response is clearly improved after addition of T helper peptides. Longer peptides can be taken up by professional APC, proteolytically processed, and then loaded onto appropriate MHC molecules and transported to the cell surface. It seems logical that the latest designed peptide vaccine should consist of multi epitopes, which could include the MHC II restricted helper epitopes recognized by CD4 T cells and MHC I restricted CD8 epitopes to induce both helper T cells and CTL, or include T and B epitopes to elicit the specific T cells and humoral responses. 

Because the antibody response to peptides is limited to the linear B cell epitopes, it would be very challenging to generate long peptides with conformations that resemble the native protein for the induction of protective antibodies [35,59]. However, the presence of antibodies that are able to recognize the peptides implies that antibodies can be induced by peptide vaccination, and might have neutralizing capacity or through enhancement of their ability to mediate CTL functions such as antibody dependent cell-mediated cytotoxicity [45,60]. New peptide based vaccines must consider promoting peptide secondary structure in order to induce a specific humoral response. Other creative and recent designs have included: engineering the distance between the repetitive epitopes on the peptides to be able of cross-linking B cell receptors for B cell proliferation; enhancing peptides antigen structure by using scaffolds; flanking by other small peptide sequences possessing propensity for folding into an α-helix; as well as adding the LI linker for better HLA presenting peptide epitopes [61,62]. Taken together, we believe it is possible that peptide vaccines can be designed to not only induce CD8 cytotoxic T lymphocytes, but also elicit an effective humoral and CD4 T cell response. 

## 4. Particulate Peptide Vaccine Delivery Methods

Peptide-based vaccines alone, even those comprising optimal B-cell and T-cell epitopes, are poorly immunogenic and require adjuvants and appropriate delivery systems to be effective. Various particulate delivery systems have been studied for efficacious delivery of peptide vaccine candidates. These delivery systems can also serve as adjuvants. Modern day particulate delivery systems are aimed at overcoming the shortcomings of the age old adjuvant, alum. The specific limitations of alum were: lack of cellular immune system stimulation, degradation on freeze drying and the possibility of adverse local reactions [2]. Particulate vaccine carriers are discussed below based on their evolution:

### 4.1. Emulsions

All emulsion based particulate carriers for vaccine are based on a common mechanism of action of formation of a depot at the injection site that is capable of attracting the immune cells. Emulsions can be single (oil-in-water (o/w), water-in-oil (w/o) or multiple (e.g., water-in-oil-in water w/o/w). The stability of emulsions as delivery systems is directly proportional to vaccine safety and efficacy; as continued presence of antigen depots at lymphoid organs releasing low-level antigens is known to stimulate a potent immune response and high-level systemic release of antigens can lead to tolerance. Their utility in delivering peptide vaccine candidates is critically determined by the process of manufacture [63], type of emulsion e.g a multiple emulsion (w/o/w) [64] the type of surfactants used to formulate the emulsion [28]. Some of the emulsion based adjuvants are discussed in Table 2.

**Table 2:** Currently marketed emulsion based delivery systems.

Example of Emulsion Based Adjuvant	Composition	Features	Encapsulated Peptide Antigen	Ref.
Freunds complete adjuvant	w/o emulsion of a mineral oil, paraffin and killed mycobacteria	Capable of generating high immunization titers Strong adverse reactions	Human papillomaviruses (HPV) E5	[65]
Montanide™ ISA 720 and 51	w/o emulsion with squalene as the oil and mono-oleate as the surfactant	Associated with adverse drug reactions; extensive and costly emulsification is needed	Human papillomaviruses (HPV) E6, E7	[65,66]
MF59™	o/w emulsion with squalene oil dispersed with the help of surfactants viz. polysorbate 80 and sorbitan oleate	Great safety profile, able to activate immune cells directly	Melanoma Peptides	[67]
AS02™	Composed of squalene and two hydrophobic immune adjuvants viz. MPL1™, a synthetic derivative of lipid A and QS-21, a purified saponin extract	Capable of inducing both humoral and cellular response	A recombinantly produced fusion of circumsporozoite protein (CS) and hepatitis B surface antigen (HBsAg), called RTS,S	[68]

A detailed literature review reveals that emulsions are a popular delivery method for various peptides. Freund’s complete adjuvant (CFA or FCA) and Freund’s incomplete adjuvant (IFA or FIA) systems are among the most popular ones to increase the humoral and cellular immunity in animals and humans. The CFA is composed of the inactivated and dried mycobacteria; whereas the IFA is devoid of the mycobacterial component. The process of emulsification is facilitated by homogenization [69] (using a homogenizer) magnetic ultrasound. Multiple emulsions have a great potential to boost the immune response because of the depot effect of the inner oil compartment and particulate adjuvant effect provided by the oil droplet. Various other modifications of existing emulsion based delivery systems have promising future, e.g., NH_2_ containing mineral oil and high purity oleic acid derivative, sorbitan monooleate. NH_2_ being a w/o emulsion was able to protect encapsulated peptide antigen from peptidase and also provided a slow release of the peptide giving sustained antigenic stimulation [70]. Similarly a novel w/o/o multiple emulsion system containing squalene oil encapsulating peptide antigen gp100 was investigated for its efficacy as a vaccine delivery system for melanoma immunotherapy [64]. The formulation was not only easily injectable, it displayed a 13 fold lower viscosity and a 6-fold (prophylactic immunization) and 2-fold (active immunization) increase in tumor growth delay time compared to w/o IFA-based gp100 peptide vaccine. Squalene oil (hydrogenated dorm of squalane oil) used in the inner compartment of the multiple emulsion does not cause any inflammatory response and is well tolerated at the site of injection. Other modifications of the emulsion system to increase the adjuvancy include DepoVax (liposome containing the adjuvant and antigen is suspended in oil), and GLA-SE (glucopyranosyl lipid adjuvant-stable emulsion), both of which are available.

### 4.2. Liposomes

Liposomes are phospholipid bilayer structures that form small vesicles mimicking cell membranes. They have an aqueous core and a lipid bilayer on the outside. They have been the most popular and extensively studied delivery vehicles for peptide antigens. The specific advantages of using liposomes as delivery vehicles for vaccine candidates are is that their composition can be tailored for biocompatibility and adaptability in order to deliver their contents to antigen presenting cells (APCs) where cross presentation is facilitated promoting cellular response. In liposomes, co-encapsulation of immunostimulants and adjuvants is possible [48]. A detailed review on the liposomes as delivery vehicles for peptide antigens is available elsewhere [48]. The composition of the phospholipid constituents in the liposomes may have an effect on the quantity and quality of the immune response produced by them, e.g., the amount of cholesterol in liposomes at and above a threshold of 20 mol % cholesterol was found to play a role in the induction of a peptide-specific immunological response in MUC1 surface decorated liposomes. Liposomes are a less preferred choice as a delivery system for peptide antigens because of their lower entrapment efficiency, although they can enhance the immunogenicity of poorly immunogenic proteins. A way to overcome this formulation issue is to conjugate the peptide moiety with lipids followed by its incorporation in liposomes. However, such incorporation was found to be variable and depended upon the nature of the lipid anchor and the lipopeptide loading [71]. Some studies wherein the antigenic peptides have been conjugated to liposomal surface have been successful. In a study by Nagata *et al.* [72], ovalbumin (OVA) conjugated on the surface of liposomes induced OVA-specific IgG antibody, not IgE antibody that is detrimental to the host. Also, they elicited CTL response in presence of CPG (added as an adjuvant) and inhibited the growth of tumors in mice [72,73]. Induction of CTL response was also observed in liposomes surface decorated with peptides derived from a non-structural polyprotein in severe acute respiratory syndrome (SARS)-associated coronavirus [70,74]. In a similar study, hepatitis C virus derived peptides coupled to surface of liposomes were found to confer complete protection to immunized mice with the establishment of long term memory [72,75]. The results obtained in this study were also replicated with OVA antigen [75] and found to produce a long lived CD8^+^ T cells without CD4^+^ T cells [73]. On the other hand, in the case of MUC1 peptides the liposome formulation with peptide displayed on the surface of liposomes were found to induce the humoral immune response [76]. The immunostimulating effect of liposomes is mediated by the protection of antigens against proteolytic enzymes. They are also known to extend the half-life of antigens in blood so that a maximum exposure of antigens to APCs occurs. Liposomes can be made positively charged (cationic liposomes), coated with polyethylene glycol (PEGs) to promote their interaction with APCs. In a study by Cortesi *et al.* [77], herpes simplex virus (HSV) peptides were encapsulated in cationic liposomes, and administered ocularly in the form of eye drops for rabbits with HSV-1 infection. A significant protection against a lethal ocular challenge was detected along with absence of reactive episodes from latency on survived animals. A size dependency in movement of cationic liposomes from the injection site to the popliteal lymph node, cell proliferation and IL-10 production was observed in a study by Henriksen-Lacey *et al.* [78], using four different sized cationic liposomes viz. (~0.2 μm, 0.6 μm, 1.5 μm and >2 μm). Cationic liposomes larger in size (>2 µm) were found to promote proliferation and low IL-10 production, whereas the liposomes of size around 500 nm were found to promote IFN-γ and IL-1B production. Liposomes can also be made pH sensitive or integrated with fusogenic peptides [79] to deliver the peptide vaccine into the cytosol and promote the CTL response. One such study involved encapsulation of CTL epitope peptides from Hantaan nucleocapsid protein (M6) or human papilloma virus E7 into pH sensitive liposomes. The liposomes were found to delay the growth of melanoma cells and the CTL response was found to be maintained for approximately 6 weeks [80]. Similar results were obtained when HIV antigen peptides were encapsulated in pH sensitive liposomes [81]. The induction of CTL is highly dependent on how effectively the antigenic peptide is delivered into the major histocompatibility complex (MHC) class I presentation pathway in APCs. Hayashi *et al.* [82] used a novel approach of adding an ER insertion signal sequence (Eriss) into the fusogenic liposomes to promote the peptide-MHC class I association for enhanced peptide transportation into the endoplasmic reticulum (ER). Such fusogenic liposomes with the Eriss signal were found to prolong the epitope presentation and enhanced induction of *in vivo* tumor immunity. Examples of liposomal particulate carriers in clinical development include AS01™ consisting of two lipophlic components viz. MPL1 and QS 21 [83]. 

### 4.3. Virosomes, ISCOMS and Related Particles

A similar group of colloids to liposomes that has been explored for the delivery of antigens are virosomes, transfersomes, archeosomes, niosomes and cochleates. Niosomes are made of non-ionic surfactants and are considered to be more stable than conventional liposome. Virosomes are composed of assembled viral membrane protein which render them enhanced binding to APCs and promote cytosolic delivery. Structurally, virosomes comprise 70% of naturally occurring phospholipids and 30% envelop phospholipids originating from the influenza virus. At the time of production the influenza surface antigens neuraminidase and hemagglutinin are integrated into the liposomal bilayer with a particle size of ~150 nm. Virosomal delivery of antigens to APCS is known to enhance MHC class I and MHC class II presentation and induce both B- and T-cell responses [84]. Virosomes are excellent adjuvant systems and are biodegradable, non-toxic, and do not induce antibodies against themselves [85]. Virosomal delivery systems are quite popular for increasing effectiveness of malarial antigens as these antigens are too small to induce any immune response [84,86]. Inflexal^®^ V is a virosomal adjuvanted Influenza vaccine (subunit) that is based on the virosome technology developed by Crucell Company (Leiden, The Netherlands), formerly Berna Biotech. It is currently registered in 43 countries for immunization against influenza for all age groups. It is also marketed under the name of InfectoVac^®^ Flu in Germany, Isiflu^®^V in Italy and Viroflu^®^ in the United Kingdom [87].

Immunostimulatory complexes (ISCOMs) are particulate antigen delivery systems composed of antigen, cholesterol, phospholipid and saponin and around 40 nm size. ISCOMATRIX™ is a particulate adjuvant comprising cholesterol, phospholipid and saponin but without antigen [88]. ISCOMs and ISCOMATRIX™ are composed of phospholipids as liposomes but also contain saponin adjuvant Quil A [89,90]. ISCOMS can only be loaded with hydrophobic antigens. Strategies to encapsulate hydrophilic antigens into ISCOMS include: coupling of antigens to ISCOMs using amphipathic coupling protein; conjugation of hydrophilic with fatty acids and phospholipids; and, modification of protein by genetic engineering. ISCOMSs are known to induce CTL responses for native as well as modified immunogens and can mediate humoral as well as cell-mediated immune responses [65,91].

### 4.4. Polymeric Particles

In recent years various polymers have been investigated for the delivery of the vaccines. The natural polymers available for the production of nanoparticles include albumin, collagen, starch, chitosan, dextran, whereas the examples of synthetic polymers include polymethylmethacrylate, polyesters, polyanhydrides, and polyamides. Of the synthetic polyesters, polylactides (PLA), polyglycolides or polyglocolic acid (PGA) and their copolymers poly(lactide-co-glycolide) PLGA are US FDA approved for use in humans and have been tested for toxicity and safety in extensive animal studies [3,92]. The popular choice for biodegradable polymers are aliphatic polyesters such as poly(lactic acid) (PLA), poly(glycolic acid) (PGA), poly(e-caprolactone) (PCL), poly(hydroxybutyrate) (PHB) and their copolymers. In particular, poly(lactide-co-glycolide) (PLGA) has been the most extensively investigated for developing nano- and microparticles encapsulating therapeutic drugs in controlled release applications. The polyesters are hydrolysed in the body generating biocompatible and metabolizable lactic acid and glycolic acid, which are removed from the body by the citric acid cycle. Owing to their particulate nature polymeric micro and nanoparticles are known to promote uptake, transport, or presentation of antigen to APCs. They were also found to elicit both cellular and humoral immunity. The biggest advantage offered by polymer based antigen delivery systems is the sustained release (for a period of few weeks to months) of the encapsulated antigen from the polymer matrix. The rate of release of the antigens from the encapsulated polymeric particles can be controlled by the rate of degradation of the polymer matrix which, in turn, is dependent on the composition of the polymer matrix, molecular weight of the polymer and size of the particles. Generally, hydrophobic interactions, electrostatic forces, hydrogen bonds, van der Waal forces, or combinations of these interactions are available as the driving forces for the formation of the polymer complexes [93]. Peptide antigens might undergo destabilization, loss of immunogenicity during the encapsulation process. Strategies to target polymeric particles to specific arms of the immune system such as the dendritic cells (DCs) in a targeted and prolonged manner have been used [48]. The encapsulation of the peptide antigens in these polymeric nanoparticles increases the longevity of the antigen to increase the possibility of its uptake by DCs. Some examples of these strategies include using charged polymers to enhance the encapsulation of the antigens and surface decoration of the particles with RGD peptide or lectins [94]. In a novel approach, long term suppression of autoimmune encephalomyelitis (EAE) was observed when peptide antigen peptide (Ac-PLP-BPI-NH2-2) was administered in hybrid nanoparticles made out of three biodegradable polymers viz. alginate-chitosan-PLGA complex when given as a one-time injection subcutaneously in mice by down regulating production of IL-6 and IL-17, cytokines associated with Th17 production [95]. 

The natural polymer, chitosan, has been known to enhance the bioavailability of the antigens due to a mucoadhesive property. A possible contributing mechanism is that chitosan has been shown to relax intercellular tight junctions and improve the paracellular transport of antigens [65]. Chitosan is a naturally occurring ideal polymer because of its nontoxic nature, biocompatibility and biodegradability. To produce a Th1 response specifically IL-12, a Th-1 inducing cytokine was added to viscous solution of chitosan and it was found to generate higher antigen-specific CD4^+^ and CD8^+^ T-cell responses [96]. Chitosan formulations were also able enhance adaptive immune response to peptide vaccines using specific antigens like GnRH-I with poor immunogenicity [97] and greater allogeneic T cell proliferation in lymph node cells of mice, and higher antigen-specific CD4^+^ and CD8^+^ T-cell responses [98]. In an interesting study by Chua *et al.* [99], the efficacy of chitosan nanoparticles of micro and nano size encapsulating LH-releasing hormone (LHRH) was investigated as an immunocontraceptive vaccine. Both sized particles were found to enhance the immunogenicity of a rather non-immunogenic LHRH without any additional adjuvant added. Despite all the merits of chitosan, one of the disadvantages is poor solubility of chitosan in water. However, researchers have identified chitosan derivatives such as 2-Hydroxypropyltrimethylammonium chloride chitosan (HACC) that is completely soluble in water.

### 4.5. Other Particulate Systems

Other particulate systems used to deliver vaccine antigens include carbon nanotubes, silicon dioxide nanoparticles, dendrimers [100], ferritin nanoparticles, peptide nanocarriers, gold nanoparticles [101], liposome-polycation-DNA (LPD) complex [102], oligosaccharide ester derivatives (OEDs) microparticles and combination systems, e.g., liposomes and w/o emulsion [92,103]. Investigators have combined the advantages of an emulsion and microparticle delivery systems in order to formulate a more efficacious delivery system. For example, PLGA microparticles were entrapped with submicron oil-in-water emulsion MF 59. *In vivo* studies on these combination systems indicate a significantly greater antibody response than individual counterparts. Also, lipid-calcium-phosphate nanoparticles are an example of multifunctional nanoparticles that are able to deliver their cargo intracellularly into the cytoplasm leading to a CTL response [49]. In an interesting study by Jiang *et al.* [104], PLGA microparticles were coated with water soluble chitosan and conjugated with M cell homing peptide (CKS9) for targeting purpose and loaded with a membrane protein B of Brachyspira hyodysenteriae (BmpB) as a model vaccine against swine dysentery. Oral immunization with these microparticle resulted in elevated secretory IgA responses in the mucosal tissues and systemic IgG antibody responses. In a similar study, PLGA microparticles were coated with chitosan or protamine to form a positive charge around them and encapsulated with CTL-restricted peptide SIINFEKL, derived from albumin. Upon single immunization of such particles in mice, the IFN-γ secreting CD8^+^ increased significantly [105]. In polyphosphazene microparticles a truncated secreted version of the F protein (ΔF) was then encapsulated with a CpG oligodeoxynucleotide (ODN) and an innate defense regulator (IDR) peptide. These microparticles, upon intramuscular or intranasal immunization, developed significantly higher levels of virus-neutralizing antibodies in the sera and lungs, and higher numbers of IFN-γ secreting cells than mice immunized with the ΔF protein alone [106]. Other examples of smart microparticles, that are sensitive to pH and release their contents in the cytosol, have been formulated with influenza A matrix protein and found to prime the CTL response [107]. 

### 4.6. Adjuvants Approved for Human Use

The first approved vaccine adjuvant was alum; it was developed by Glenny *et al.* [5] in 1926 with diphtheria toxoid absorbed to alum, and has been used for over eight decades. In 1936, Freund developed an emulsion of water and mineral oil including killed mycobacteria, known as Freund’s complete adjuvant (FCA), a gold standard adjuvant for efficacy [83]. However, FCA induces severe local necrotic ulcers, and is considered too toxic for human use. Without added mycobacteria, this oil-based adjuvant is known as Freund’s incomplete adjuvant (IFA), and is less toxic and used for human vaccine formulation [5]. More bacterial components have been studied as adjuvants, after lipopolysaccharides (LPS) and lipophilic phospholipid (lipid A) were found to exhibit adjuvant activity [52]. A number of natural and synthetic compounds have been demonstrated to have adjuvant activity; unfortunately many of them are more toxic for human use when compared to alum. There are several new approvals for human vaccines from FDA and Europe such as MF59, virosome, AS03, AF03, and AS04 (monphosphoryl lipid A (MPL) with alum. MF59 is composed of squalene, was produced by Novartis vaccines and diagnostics Inc. It presents as an adjuvant component of influenza vaccine for elderly patients (Fluad^®^) [108,109,110]. Virosome, consisting of unilamellar phospholipid membrane (reconstituted empty influenza virus envelopes), was licensed for Inflexal^®^ V, Invivac^®^ influenza vaccine, and hepatitis A vaccines (Epaxal^®^). AS03 is the trade name for a squalene-based immunologic adjuvant, which was licensed for pandemic flu vaccine (Pandemrix^®^) by GlaxoSmithKline [52]. AF03 is also a squalene-based emulsion adjuvant that is present in the adjuvanted pandemic influenza vaccine, Humenza™ [83]. AS04 was also developed by GSK and used as the adjuvant for viral vaccines including hepatitis B (Fendrix^®^), and HPV (Cervarix^®^) [111]. AS04 is comprised of traditional alum with a Toll-like receptor (TLR) agonist. MPL adjuvant is a chemically modified derivative of lipopolysaccharide that displays greatly reduced toxicity while maintaining most of the immunostimulatory activity of lipopolysaccharide; it is a potent stimulator of T cell and antibody responses. The wide ranges of clinical trials carried out on MPL have resulted in over 33,000 doses being administered to over 12,000 individuals [112]. MPL is also the first and only TLR ligand in licensed human vaccines, in the form of AS04. With a history of safe and effective use in licensed vaccines, such technologies can now be considered for particulate peptide vaccine delivery. 

### 4.7. Safety Issues with Particulate Delivery Strategies

Safety of the particulate delivery strategies is of highest concern when selecting the particular strategies for delivering the peptide antigen. The route of administration from which the particulate delivery system is administered plays a vital role in toxicity determination. The common routes of administration of the vaccines are subcutaneous, intranasal, intravenous, and transdermal. Of these, the transdermal route has been the safest and meritorious in terms of: being needle free, therefore eliminating the need for specially trained health-care workers to administer vaccines; lower cost; ability to confer mucosal as well as systemic immunity; increased stability; increased shelf life [113]. For the polymeric particulate delivery system, the hydrophobicity of the polymer [114] and PEGylation on the polymer [115] can play a huge role determining the biodistribution, elimination and, hence, the toxicity. Hydrophobicity of the polymer determines the membrane disruptive property and therefore toxicity of the polymers. The polymers investigated by Shima *et al.* [114], showed membrane disruptive activity only at the endosomal pH range which is required for endosomal escape of the entrapped antigen and cytosolic delivery of the antigen for inducing the CTL immune response. PEGylation of the polymer makes it more hydrophilic, *i.e.*, reducing the hydrophobicity, whereas decreasing distribution in liver and spleen as time indicated nanoparticles are broken down and cleared, increasing its safety [115]. Poly (gamma-glutamic acid) nanoparticulate systems have been studied for any histopathological changes after subcutaneous injection, or acute toxicity after intravenous injection, and they were found to be safe. Safety of the vaccines delivered via the particulate delivery system is usually established by giving repeated administration of the vaccine at therapeutic or elevated doses as per certain guidelines e.g., OECD or WHO [116]. The criteria that is used to evaluate safety may include immediate adverse events (AE) reported within 30 min of vaccination; solicited and unsolicited AEs, serious AEs and clinical laboratory safety measurements [117]. 

## 5. Summary

Vaccine development is a complex and challenging process. Peptide vaccines provide several advantages in comparison to conventional vaccines. Peptide vaccines are a safe and economical technology compared to traditional vaccines made of dead or attenuated pathogens, inactivated toxins, and recombinant subunits. Peptide vaccine production is relatively inexpensive due to the ease of production and simplistic composition. Additionally, peptide vaccines avoid the inclusion of unnecessary components possessing high reactogenicity to the host, such as lipopolysaccharides, lipids, and toxins. Moreover, peptide vaccines can be composed of various epitopes from different antigens, and integrate T cells and B cells epitopes into one antigenic formulation. Despite several advantages, peptides are typically poorly immunogenic when used alone, requiring the next generation of adjuvants to overcome this problem. Future peptide vaccine delivery strategies may target the combination of adjuvants in formulations, strategies to enhance the ability to enhance antigen uptake, novel adjuvants to stimulate innate immunity and induce specific adaptive immune response. However, the safety of adjuvants themselves remains a major concern. If one could devise means to overcome the safety issues related to the use of adjuvants and particulate peptide vaccine delivery systems, peptide vaccine technology has the potential to grow into the next generation of subunit vaccines.

## References

[1] Bachler B.C., Humbert M., Palikuqi B., Siddappa N.B., Lakhashe S.K., Rasmussen R.A., Ruprecht R.M. (2013). Novel biopanning strategy to identify epitopes associated with vaccine protection. J. Virol..

[2] Perrie Y., Kirby D., Bramwell V.W., Mohammed A.R. (2007). Recent developments in particulate-based vaccines. Recent Pat. Drug Deliv. Formul..

[3] Black M., Trent A., Tirrell M., Olive C. (2010). Advances in the design and delivery of peptide subunit vaccines with a focus on toll-like receptor agonists. Expert Rev. Vaccines.

[4] Thompson A.L., Staats H.F. (2011). Cytokines: The future of intranasal vaccine adjuvants. Clin. Dev. Immunol..

[5] Petrovsky N., Aguilar J.C. (2004). Vaccine adjuvants: Current state and future trends. Immunol. Cell Biol..

[6] Sesardic D. (1993). Synthetic peptide vaccines. J. Med. Microbiol..

[7] Bijker M.S., Melief C.J., Offringa R., van der Burg S.H. (2007). Design and development of synthetic peptide vaccines: Past, present and future. Expert Rev. Vaccines.

[8] Lin S.Y., Cheng C.W., Su E.C. (2013). Prediction of B-cell epitopes using evolutionary information and propensity scales. BMC Bioinform..

[9] Purcell A.W., McCluskey J., Rossjohn J. (2007). More than one reason to rethink the use of peptides in vaccine design. Nat. Rev. Drug Discov..

[10] Aguilar J.C., Rodriguez E.G. (2007). Vaccine adjuvants revisited. Vaccine.

[11] Liu Y., McNevin J., Zhao H., Tebit D.M., Troyer R.M., McSweyn M., Ghosh A.K., Shriner D., Arts E.J., McElrath M.J. (2007). Evolution of human immunodeficiency virus type 1 cytotoxic T-lymphocyte epitopes: Fitness-balanced escape. J. Virol..

[12] Kolesanova E.F., Sanzhakov M.A., Kharybin O.N. (2013). Development of the schedule for multiple parallel “difficult” Peptide synthesis on pins. Int. J. Pept..

[13] Epstein J.E., Giersing B., Mullen G., Moorthy V., Richie T.L. (2007). Malaria vaccines: Are we getting closer?. Curr. Opin. Mol. Ther..

[14] Volpina O.M., Gelfanov V.M., Yarov A.V., Surovoy A.Y., Chepurkin A.V., Ivanov V.T. (1993). New virus-specific T-helper epitopes of foot-and-mouth disease viral VP1 protein. FEBS Lett..

[15] Tarradas J., Monso M., Munoz M., Rosell R., Fraile L., Frías M.T., Domingo M., Andreu D., Sobrino F., Ganges L. (2011). Partial protection against classical swine fever virus elicited by dendrimeric vaccine-candidate peptides in domestic pigs. Vaccine.

[16] Stanekova Z., Kiraly J., Stropkovska A., Mikušková T., Mucha V., Kostolanský F., Varečková E. (2011). Heterosubtypic protective immunity against influenza a virus induced by fusion peptide of the hemagglutinin in comparison to ectodomain of M2 protein. Acta Virol..

[17] Oscherwitz J., Yu F., Cease K.B. (2010). A synthetic peptide vaccine directed against the 2ss2–2ss3 loop of domain 2 of protective antigen protects rabbits from inhalation anthrax. J. Immunol..

[18] Solares A.M., Baladron I., Ramos T., Valenzuela C., Borbon Z., Fanjull S., Gonzalez L., Castillo D., Esmir J., Granadillo M. (2011). Safety and immunogenicity of a human papillomavirus peptide vaccine (CIGB-228) in women with high-grade cervical intraepithelial neoplasia: first-in-human, proof-of-concept trial. ISRN Obstet. Gynecol..

[19] Bernhardt S.L., Gjertsen M.K., Trachsel S., Møller M., Eriksen J.A., Meo M., Buanes T., Gaudernack G. (2006). Telomerase peptide vaccination of patients with non-resectable pancreatic cancer: A dose escalating phase I/II study. Br. J. Cancer.

[20] Brunsvig P.F., Aamdal S., Gjertsen M.K., Kvalheim G., Markowski-Grimsrud C.J., Sve I., Dyrhaug M., Trachsel S., Møller M., Eriksen J.A. (2006). Telomerase peptide vaccination: A phase I/II study in patients with non-small cell lung cancer. Cancer Immunol. Immunother..

[21] Brunsvig P.F., Kyte J.A., Kersten C., Sundstrøm S., Møller M., Nyakas M., Hansen G.L., Gaudernack G., Aamdal S. (2011). Telomerase peptide vaccination in NSCLC: A phase II trial in stage III patients vaccinated after chemoradiotherapy and an 8-year update on a phase I/II trial. Clin. Cancer Res..

[22] Kyte J.A., Gaudernack G., Dueland S., Trachsel S., Julsrud L., Aamdal S. (2011). Telomerase peptide vaccination combined with temozolomide: A clinical trial in stage IV melanoma patients. Clin. Cancer Res..

[23] Greten T.F., Forner A., Korangy F., N’Kontchou G., Barget N., Ayuso C., Ormandy L.A., Manns M.P., Beaugrand M., Bruix J. (2010). A phase II open label trial evaluating safety and efficacy of a telomerase peptide vaccination in patients with advanced hepatocellular carcinoma. BMC Cancer.

[24] Kyte J.A., Trachsel S., Risberg B., Thor S.P., Lislerud K., Gaudernack G. (2009). Unconventional cytokine profiles and development of T cell memory in long-term survivors after cancer vaccination. Cancer Immunol. Immunother..

[25] Demento S.L., Siefert A.L., Bandyopadhyay A., Sharp F.A., Fahmy T.M. (2011). Pathogen-associated molecular patterns on biomaterials: A paradigm for engineering new vaccines. Trends Biotechnol..

[26] Burioni R., Perotti M., Mancini N., Clementi M. (2008). Perspectives for the utilization of neutralizing human monoclonal antibodies as anti-HCV drugs. J. Hepatol..

[27] Testa J.S., Philip R. (2012). Role of T-cell epitope-based vaccine in prophylactic and therapeutic applications. Future Virol..

[28] Lanier J.G., Newman M.J., Lee E.M., Sette A., Ahmed R. (1999). Peptide vaccination using nonionic block copolymers induces protective anti-viral CTL responses. Vaccine.

[29] Corti D., Voss J., Gamblin S.J., Codoni G., Macagno A., Jarrossay D., Vachieri S.G., Pinna D., Minola A., Vanzetta F. (2011). A neutralizing antibody selected from plasma cells that binds to group 1 and group 2 influenza A hemagglutinins. Science.

[30] Hager-Braun C., Tomer K.B. (2005). Determination of protein-derived epitopes by mass spectrometry. Expert Rev. Proteomics.

[31] Panina-Bordignon P., Demotz S., Corradin G., Lanzavecchia A. (1989). Study on the immunogenicity of human class-II-restricted T-cell epitopes: Processing constraints, degenerate binding, and promiscuous recognition. Cold Spring Harb. Symp. Quant. Biol..

[32] Huang J., Ru B., Zhu P., Nie F., Yang J., Wang X., Dai P., Lin H., Guo F.B., Rao N. (2012). MimoDB 2.0: A mimotope database and beyond. Nucleic Acids Res..

[33] Gershoni J.M., Roitburd-Berman A., Siman-Tov D.D., Tarnovitski F.N., Weiss Y. (2007). Epitope mapping: The first step in developing epitope-based vaccines. BioDrugs.

[34] Langeveld J.P., Casal J.I., Osterhaus A.D., Cortés E., de Swart R., Vela C., Dalsgaard K., Puijk W.C., Schaaper W.M., Meloen R.H. (1994). First peptdie vaccine providing protection against viral infection in the target animal; studies of canine parvovirus in dogs. J. Virol..

[35] Kupriianova M.A., Zhmak M.N., Koroev D.O., Chepurkin A.V., Vol’pina O.M., Ivanov V.T. (2000). Synthetic peptide designs based on immunoactive fragments of the VP1 protein of the foot-and-mouth disease virus strain A22. Bioorg. Khimiia.

[36] Robinson J., Waller M.J., Fail S.C., Mcwilliam H., Lopez R., Parham P., Marsh S.G.E. (2009). The IMGT/HLA database. Nucleic Acids Res..

[37] Schuler M.M., Nastke M.D., Stevanovikc S. (2007). SYFPEITHI: Database for searching and T-cell epitope prediction. Methods Mol. Biol..

[38] Michielin O., Luescher I., Karplus M. (2000). Modeling of the TCR-MHC-peptide complex. J. Mol. Biol..

[39] Lin H.H., Ray S., Tongchusak S., Reinherz E.L., Brusic V. (2008). Evaluation of MHC class I peptide binding prediction servers: Applications for vaccine research. BMC Immunol..

[40] Townsend A.R., Rothbard J., Gotch F.M., Bahadur G., Wraith D., McMichael A.J. (1986). The epitopes of influenza nucleoprotein recognized by cytotoxic T lymphocytes can be defined with short synthetic peptides. Cell.

[41] Townsend A.R., McMichael A.J. (1985). Specificity of cytotoxic T lymphocytes stimulated with influenza virus. Studies in mice and humans. Prog. Allergy.

[42] Townsend A.R., Skehel J.J., Taylor P.M., Palese P. (1984). Recognition of influenza A virus nucleoprotein by an H-2-restricted cytotoxic T-cell clone. Virology.

[43] Aichele P., Brduscha-Riem K., Zinkernagel R.M., Hengartner H., Pircher H. (1995). T cell priming *versus* T cell tolerance induced by synthetic peptides. J. Exp. Med..

[44] Aichele P., Hengartner H., Zinkernagel R.M., Schulz M. (1990). Antiviral cytotoxic T cell response induced by *in vivo* priming with a free synthetic peptide. J. Exp. Med..

[45] Azizi A., Anderson D.E., Torres J.V., Ogrel A., Ghorbani M., Soare C., Sandstrom P., Fournier J., Diaz-Mitoma F. (2008). Induction of broad cross-subtype-specific HIV-1 immune responses by a novel multivalent HIV-1 peptide vaccine in cynomolgus macaques. J. Immunol..

[46] Kast W.M., Roux L., Curren J., Blom H.J., Voordouw A.C., Meloen R.H., Kolakofsky D., Melief C.J. (1991). Protection against lethal Sendai virus infection by *in vivo* priming of virus-specific cytotoxic T lymphocytes with a free synthetic peptide. Proc. Natl. Acad. Sci. USA.

[47] Wahala W.M., Silva A.M. (2011). The human antibody response to dengue virus infection. Viruses.

[48] Joshi M.D., Unger W.J., Storm G., van K.Y., Mastrobattista E. (2012). Targeting tumor antigens to dendritic cells using particulate carriers. J. Control Release.

[49] Xu Z., Ramishetti S., Tseng Y.C., Guo S., Wang Y., Huang L. (2013). Multifunctional nanoparticles co-delivering Trp2 peptide and CpG adjuvant induce potent cytotoxic T-lymphocyte response against melanoma and its lung metastasis. J. Control Release.

[50] Quakkelaar E.D., Melief C.J. (2012). Experience with synthetic vaccines for cancer and persistent virus infections in nonhuman primates and patients. Adv. Immunol..

[51] Quakkelaar E.D., Bunnik E.M., van Alphen F.P., Boeser-Nunnink B.D., van Nuenen A.C., Schuitemaker H. (2007). Escape of human immunodeficiency virus type 1 from broadly neutralizing antibodies is not associated with a reduction of viral replicative capacity *in vitro*. Virology.

[52] Moisa A.A., Kolesanova E.F. (2011). Synthetic peptide vaccines. Biomed. Khimiia.

[53] Fayolle C., bdel-Motal U.M., Berg L., Deriaud E., Jondal M., Leclerc C. (1996). Induction of cytotoxic T-cell response by optimal-length peptides does not require CD4^+^ T-cell help. Immunology.

[54] Fayolle C., Sebo P., Ladant D., Ullmann A., Leclerc C. (1996). *In vivo* induction of CTL responses by recombinant adenylate cyclase of Bordetella pertussis carrying viral CD8^+^ T cell epitopes. J. Immunol..

[55] Zwaveling S., Ferreira Mota S.C., Nouta J., Johnson M., Lipford G.B., Offringa R., van der Burg S.H., Melief C.J. (2002). Established human papillomavirus type 16-expressing tumors are effectively eradicated following vaccination with long peptides. J. Immunol..

[56] Welters M.J., Kenter G.G., Piersma S.J., Vloon A.P., Löwik M.J., Berends-van der Meer D.M., Drijfhout J.W., Valentijn A.R., Wafelman A.R., Oostendorp J. (2008). Induction of tumor-specific CD4^+^ and CD8^+^ T-cell immunity in cervical cancer patients by a human papillomavirus type 16 E6 and E7 long peptides vaccine. Clin. Cancer Res..

[57] Welters M.J., van der L.P., van den Eeden S.J., Kwappenberg K.M.C., Drijfhout J.W., Fleuren G.J., Kenter G.G., Melief C.J.M., van der Burg S.H., Offringa R. (2006). Detection of human papillomavirus type 18 E6 and E7-specific CD4^+^ T-helper 1 immunity in relation to health *versus* disease. Int. J. Cancer.

[58] Welters M.J., Kenter G.G., de Vos van Steenwijk P.J., Löwik M.J., Berends-van der Meer D.M., Essahsah F., Stynenbosch L.F., Vloon A.P., Ramwadhdoebe T.H., Piersma S.J. (2010). Success or failure of vaccination for HPV16-positive vulvar lesions correlates with kinetics and phenotype of induced T-cell responses. Proc. Natl. Acad. Sci. USA.

[59] Panina-Bordignon P., Tan A., Termijtelen A., Demotz S., Corradin G., Lanzavecchia A. (1989). Universally immunogenic T cell epitopes: Promiscuous binding to human MHC class II and promiscuous recognition by T cells. Eur. J. Immunol..

[60] Agadjanyan M.G., Ghochikyan A., Petrushina I., Vasilevko V., Movsesyan N., Mkrtichyan M., Saing T., Cribbs D.H. (2005). Prototype Alzheimer’s disease vaccine using the immunodominant B cell epitope from beta-amyloid and promiscuous T cell epitope pan HLA DR-binding peptide. J. Immunol..

[61] Chen W., Ede N.J., Jackson D.C., McCluskey J., Purcell A.W. (1996). CTL recognition of an altered peptide associated with asparagine bond rearrangement. Implications for immunity and vaccine design. J. Immunol..

[62] Jackson D.C., Purcell A.W., Fitzmaurice C.J., Zeng W., Hart D.N. (2002). The central role played by peptides in the immune response and the design of peptide-based vaccines against infectious diseases and cancer. Curr. Drug Targets.

[63] Koh Y.T., Higgins S.A., Weber J.S., Kast W.M. (2006). Immunological consequences of using three different clinical/laboratory techniques of emulsifying peptide-based vaccines in incomplete Freund’s adjuvant. J. Transl. Med..

[64] Kalariya M., Ganta S., Amiji M. (2012). Multi-compartmental vaccine delivery system for enhanced immune response to gp100 peptide antigen in melanoma immunotherapy. Pharm. Res..

[65] Chen Y.F., Lin C.W., Tsao Y.P., Chen S.L. (2004). Cytotoxic-T-lymphocyte human papillomavirus type 16 E5 peptide with CpG-oligodeoxynucleotide can eliminate tumor growth in C57BL/6 mice. J. Virol..

[66] Van Driel W.J., Ressing M.E., Kenter G.G., Brandt R.M., Krul E.J., van Rossum A.B., Schuuring E., Offringa R., Bauknecht T., Tamm-Hermelink A. (1999). Vaccination with HPV16 peptides of patients with advanced cervical carcinoma: Clinical evaluation of a phase I–II trial. Eur. J. Cancer.

[67] Slingluff C.L., Petroni G.R., Yamshchikov G.V., Barnd D.L., Eastham S., Galavotti H., Patterson J.W., Deacon D.H., Hibbitts S., Teates D. (2003). Clinical and immunologic results of a randomized phase II trial of vaccination using four melanoma peptides either administered in granulocyte-macrophage colony-stimulating factor in adjuvant or pulsed on dendritic cells. J. Clin. Oncol..

[68] Pinder M., Reece W.H., Plebanski M., Akinwunmi P., Flanagan K.L., Lee E.A., Doherty T., Milligan P., Jaye A., Tornieporth N. (2004). Cellular immunity induced by the recombinant Plasmodium falciparum malaria vaccine, RTS,S/AS02, in semi-immune adults in The Gambia. Clin. Exp. Immunol..

[69] Beebe M., Qin M., Moi M., Wu S., Heiati H., Walker L., Newman M., Fikes J., Ishioka G.Y. (2008). Formulation and characterization of a ten-peptide single-vial vaccine, EP-2101, designed to induce cytotoxic T-lymphocyte responses for cancer immunotherapy. Hum. Vaccines.

[70] Iseki K., Matsunaga H., Komatsu N., Suekane S., Noguchi M., Itoh K., Yamada A. (2010). Evaluation of a new oil adjuvant for use in peptide-based cancer vaccination. Cancer Sci..

[71] Liang M.T., Davies N.M., Blanchfield J.T., Toth I. (2006). Particulate systems as adjuvants and carriers for peptide and protein antigens. Curr. Drug Deliv..

[72] Nagata T., Toyota T., Ishigaki H., Ichihashi T., Kajino K., Kashima Y., Itoh Y., Mori M., Oda H., Yamamura H. (2007). Peptides coupled to the surface of a kind of liposome protect infection of influenza viruses. Vaccine.

[73] Taneichi M., Tanaka Y., Kakiuchi T., Uchida T. (2010). Liposome-coupled peptides induce long-lived memory CD8 T cells without CD4 T cells. PLoS One.

[74] Kohyama S., Ohno S., Suda T., Taneichi M., Yokoyama S., Mori M., Kobayashi A., Hayashi H., Uchida T., Matsui M. (2009). Efficient induction of cytotoxic T lymphocytes specific for severe acute respiratory syndrome (SARS)-associated coronavirus by immunization with surface-linked liposomal peptides derived from a non-structural polyprotein 1a. Antivir. Res..

[75] Takagi A., Matsui M., Ohno S., Duan H., Moriya O., Kobayashi N., Oda H., Mori M., Kobayashi A., Taneichi M. (2009). Highly efficient antiviral CD8^+^ T-cell induction by peptides coupled to the surfaces of liposomes. Clin. Vaccine Immunol..

[76] Guan H.H., Budzynski W., Koganty R.R., Krantz M.J., Reddish M.A., Rogers J.A., Longenecker B.M., Samuel J. (1998). Liposomal formulations of synthetic MUC1 peptides: Effects of encapsulation *versus* surface display of peptides on immune responses. Bioconjug. Chem..

[77] Cortesi R., Argnani R., Esposito E., Dalpiaz A., Scatturin A., Bortolotti F., Lufino M., Guerrini R., Cavicchioni G., Incorvaia C. (2006). Cationic liposomes as potential carriers for ocular administration of peptides with anti-herpetic activity. Int. J. Pharm..

[78] Henriksen-Lacey M., Korsholm K.S., Andersen P., Perrie Y., Christensen D. (2011). Liposomal vaccine delivery systems. Expert Opin. Drug Deliv..

[79] Sugita T., Yoshikawa T., Gao J.Q., Shimokawa M., Oda A., Niwa T., Akashi M., Tsutsumi Y., Mayumi T., Nakagawa S. (2005). Fusogenic liposome can be used as an effective vaccine carrier for peptide vaccination to induce cytotoxic T lymphocyte (CTL) response. Biol. Pharm. Bull..

[80] Chang J.S., Choi M.J., Cheong H.S., Kim K. (2001). Development of Th1-mediated CD8^+^ effector T cells by vaccination with epitope peptides encapsulated in pH-sensitive liposomes. Vaccine.

[81] Chang J.S., Choi M.J., Kim T.Y., Cho S.Y., Cheong H.S. (1999). Immunogenicity of synthetic HIV-1 V3 loop peptides by MPL adjuvanted pH-sensitive liposomes. Vaccine.

[82] Hayashi A., Wakita H., Yoshikawa T., Nakanishi T., Tsutsumi Y., Mayumie T., Mukai Y., Yoshioka Y., Okada N., Nakagawa S. (2007). A strategy for efficient cross-presentation of CTL-epitope peptides leading to enhanced induction of *in vivo* tumor immunity. J. Control Release.

[83] Awate S., Babiuk L.A., Mutwiri G. (2013). Mechanisms of action of adjuvants. Front. Immunol..

[84] Okitsu S.L., Boato F., Mueller M.S., Li D.B., Vogel D., Westerfeld N., Zurbriggen R., Robinson J.A., Pluschke G. (2007). Antibodies elicited by a virosomally formulated *Plasmodium falciparum* serine repeat antigen-5 derived peptide detect the processed 47 kDa fragment both in sporozoites and merozoites. Peptides.

[85] Cryz S.J., Que J.U., Gluck R. (1996). A virosome vaccine antigen delivery system does not stimulate an antiphospholipid antibody response in humans. Vaccine.

[86] Westerfeld N., Pluschke G., Zurbriggen R. (2006). Optimized Malaria-antigens delivered by immunostimulating reconstituted influenza virosomes. Wien. klinische Wochenschr..

[87] Herzog C., Hartmann K., Kunzi V., Kürsteiner O., Mischler R., Lazar H., Glück R. (2009). Eleven years of inflexal V-a virosomal adjuvanted influenza vaccine. Vaccine.

[88] Sun H.X., Xie Y., Ye Y.P. (2009). ISCOMs and ISCOMATRIX. Vaccine.

[89] Morelli A.B., Becher D., Koernig S., Silva A., Drane D., Maraskovsky E. (2012). ISCOMATRIX: A novel adjuvant for use in prophylactic and therapeutic vaccines against infectious diseases. J. Med. Microbiol..

[90] Duewell P., Kisser U., Heckelsmiller K., Hoves S., Stoitzner P., Koernig S., Morelli A.B., Clausen B.E., Dauer M., Eigler A. (2011). ISCOMATRIX adjuvant combines immune activation with antigen delivery to dendritic cells *in vivo* leading to effective cross-priming of CD8^+^ T cells. J. Immunol..

[91] Agrawal L., Haq W., Hanson C.V., Rao D.N. (2003). Generating neutralizing antibodies, Th1 response and MHC non restricted immunogenicity of HIV-I env and gag peptides in liposomes and ISCOMs with in-built adjuvanticity. J. Immune Based Ther. Vaccines.

[92] Reed S.G., Orr M.T., Fox C.B. (2013). Key roles of adjuvants in modern vaccines. Nat. Med..

[93] Kang N., Perron M.E., Prud’homme R.E., Zhang Y., Gaucher G., Leroux J.C. (2005). Stereocomplex block copolymer micelles: Core-shell nanostructures with enhanced stability. Nano Lett..

[94] Correia-Pinto J.F., Csaba N., Alonso M.J. (2013). Vaccine delivery carriers: Insights and future perspectives. Int. J. Pharm..

[95] Buyuktimkin B., Wang Q., Kiptoo P., Stewart J.M., Berkland C., Siahaan T.J. (2012). Vaccine-like controlled-release delivery of an immunomodulating peptide to treat experimental autoimmune encephalomyelitis. Mol. Pharm..

[96] Heffernan M.J., Zaharoff D.A., Fallon J.K., Schlom J., Greiner J.W. (2011). *In vivo* efficacy of a chitosan/IL-12 adjuvant system for protein-based vaccines. Biomaterials.

[97] Saenz L., Neira-Carrillo A., Paredes R., Cortes M., Bucarey S., Arias J.L. (2009). Chitosan formulations improve the immunogenicity of a GnRH-I peptide-based vaccine. Int. J. Pharm..

[98] Zaharoff D.A., Rogers C.J., Hance K.W., Schlom J., Greiner J.W. (2007). Chitosan solution enhances the immunoadjuvant properties of GM-CSF. Vaccine.

[99] Chua B.Y., Al K.M., Zeng W., Mainwaring D., Jackson D.C. (2012). Chitosan microparticles and nanoparticles as biocompatible delivery vehicles for peptide and protein-based immunocontraceptive vaccines. Mol. Pharm..

[100] Gaertner H.F., Cerini F., Kamath A., Rochat A.-F., Siegrist C.-A., Menin L., Hartley O. (2011). Efficient orthogonal bioconjugation of dendrimers for synthesis of bioactive nanoparticles. Bioconjug. Chem..

[101] Chen Y.S., Hung Y.C., Lin L.W., Liau I., Hong M.Y., Huang G.S. (2010). Size-dependent impairment of cognition in mice caused by the injection of gold nanoparticles. Nanotechnology.

[102] Jalali S.A., Sankian M., Tavakkol-Afshari J., Jaafari M.R. (2012). Induction of tumor-specific immunity by multi-epitope rat HER2/neu-derived peptides encapsulated in LPD Nanoparticles. Nanomedicine.

[103] Daftarian P., Mansour M., Benoit A.C., Pohajdak B., Hoskin D.W., Brown R.G., Kast W.M. (2006). Eradication of established HPV 16-expressing tumors by a single administration of a vaccine composed of a liposome-encapsulated CTL-T helper fusion peptide in a water-in-oil emulsion. Vaccine.

[104] Jiang T., Singh B., Li H.S., Kim Y.K., Kang S.K., Nah J.W., Choi Y.J., Cho C.S. (2014). Targeted oral delivery of BmpB vaccine using porous PLGA microparticles coated with M cell homing peptide-coupled chitosan. Biomaterials.

[105] Fischer S., Schlosser E., Mueller M., Csaba N., Merkle H.P., Groettrup M., Gander B. (2009). Concomitant delivery of a CTL-restricted peptide antigen and CpG ODN by PLGA microparticles induces cellular immune response. J. Drug Target..

[106] Garlapati S., Garg R., Brownlie R., Latimer L., Simko E., Hancock R.E., Babiuk L.A., Gerdts V., Potter A., van Drunen Littel-van den Hurk S. (2012). Enhanced immune responses and protection by vaccination with respiratory syncytial virus fusion protein formulated with CpG oligodeoxynucleotide and innate defense regulator peptide in polyphosphazene microparticles. Vaccine.

[107] Haining W.N., Anderson D.G., Little S.R., von Bergwelt-Baildon M.S., Cardoso A.A., Alves P., Kosmatopoulos K., Nadler L.M., Langer R., Kohane D.S. (2004). pH-triggered microparticles for peptide vaccination. J. Immunol..

[108] Seubert A., Calabro S., Santini L., Galli B., Genovese A., Valentini S., Aprea S., Colaprico A., D’Oro U., Giuliani M.M. (2011). Adjuvanticity of the oil-in-water emulsion MF59 is independent of Nlrp3 inflammasome but requires the adaptor protein MyD88. Proc. Natl. Acad. Sci. USA.

[109] Calabro S., Tortoli M., Baudner B.C., Pacitto A., Cortese M., O’Hagan D.T., de Gregorio E., Seubert A., Wack A. (2011). Vaccine adjuvants alum and MF59 induce rapid recruitment of neutrophils and monocytes that participate in antigen transport to draining lymph nodes. Vaccine.

[110] Dupuis M., McDonald D.M., Ott G. (1999). Distribution of adjuvant MF59 and antigen gD2 after intramuscular injection in mice. Vaccine.

[111] Didierlaurent A.M., Morel S., Lockman L., Giannini S.L., Bisteau M., Carlsen H., Kielland A., Vosters O., Vanderheyde N., Schiavetti F. (2009). AS04, an aluminum salt- and TLR4 agonist-based adjuvant system, induces a transient localized innate immune response leading to enhanced adaptive immunity. J. Immunol..

[112] Baldrick P., Richardson D., Elliott G., Wheeler A.W. (2002). Safety evaluation of monophosphoryl lipid A (MPL): An immunostimulatory adjuvant. Regul. Toxicol. Pharmacol..

[113] Lawson L.B., Freytag L.C., Clements J.D. (2007). Use of nanocarriers for transdermal vaccine delivery. Clin. Pharmacol. Ther..

[114] Shima F., Akagi T., Akashi M. (2014). The role of hydrophobicity in the disruption of erythrocyte membrane by nanoparticles composed of hydrophobically modified poly(gamma-glutamic acid). J. Biomater. Sci. Polym. Ed..

[115] Yang Y., Neef T., Mittelholzer C., Garcia Garayoa E., Bläuenstein P., Schibli R., Aebi U., Burkhard P. (2013). The biodistribution of self-assembling protein nanoparticles shows they are promising vaccine platforms. J. Nanobiotechnol..

[116] WHO Vaccine Standardization. http://www.who.int/biologicals/publications/nonclinical_evaluation_vaccines_nov_2003.pdf.

[117] Glenn G.M., Smith G., Fries L., Raghunandan R., Lu H., Zhou B., Thomas D.N., Hickman S.P., Kpamegan E., Boddapati S. (2013). Safety and immunogenicity of a Sf9 insect cell-derived respiratory syncytial virus fusion protein nanoparticle vaccine. Vaccine.

